# Glial response during cuprizone-induced de- and remyelination in the CNS: lessons learned

**DOI:** 10.3389/fncel.2014.00073

**Published:** 2014-03-13

**Authors:** Viktoria Gudi, Stefan Gingele, Thomas Skripuletz, Martin Stangel

**Affiliations:** ^1^Department of Neurology, Hannover Medical SchoolHannover, Germany; ^2^Center for Systems NeuroscienceHannover, Germany

**Keywords:** cuprizone mouse model, remyelination, demyelination, growth factors, microglia, astrocytes

## Abstract

Although astrogliosis and microglia activation are characteristic features of multiple sclerosis (MS) and other central nervous system (CNS) lesions the exact functions of these events are not fully understood. Animal models help to understand the complex interplay between the different cell types of the CNS and uncover general mechanisms of damage and repair of myelin sheaths. The so called cuprizone model is a toxic model of demyelination in the CNS white and gray matter, which lacks an autoimmune component. Cuprizone induces apoptosis of mature oligodendrocytes that leads to a robust demyelination and profound activation of both astrocytes and microglia with regional heterogeneity between different white and gray matter regions. Although not suitable to study autoimmune mediated demyelination, this model is extremely helpful to elucidate basic cellular and molecular mechanisms during de- and particularly remyelination independently of interactions with peripheral immune cells. Phagocytosis and removal of damaged myelin seems to be one of the major roles of microglia in this model and it is well known that removal of myelin debris is a prerequisite of successful remyelination. Furthermore, microglia provide several signals that support remyelination. The role of astrocytes during de- and remyelination is not well defined. Both supportive and destructive functions have been suggested. Using the cuprizone model we could demonstrate that there is an important crosstalk between astrocytes and microglia. In this review we focus on the role of glial reactions and interaction in the cuprizone model. Advantages and limitations of as well as its potential therapeutic relevance for the human disease MS are critically discussed in comparison to other animal models.

## Introduction

**Figure 1:**
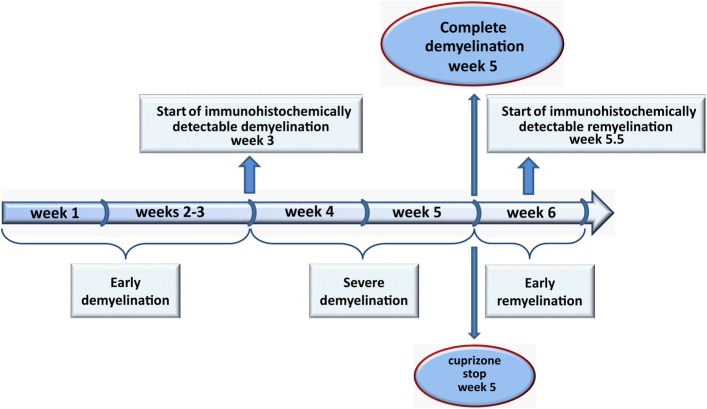
**The course of de- and remyelination in the medial corpus callosum.** 8–10 weeks old C57BL/6 mice were fed with 0.2% (w/w) cuprizone for 5 weeks. During the “early demyelination” (1–3 weeks) the degradation of myelin proteins begins and can be hardly detectable using immunohistochemical techniques. After week 3 onwards demyelination progresses (“severe demyelination”) and is complete at week 5 (“complete demyelination”). Remyelination starts immediately thereafter. Between weeks 5 and 6 numerous remyelinated axons appear in the medial corpus callosum (“early remyelination”). We therefore, suggest to stop the cuprizone administration after 5 weeks to allow newly generated oligodendrocytes to restore myelin sheaths without any influence of cuprizone.

Demyelination of the central nervous system (CNS) is the hallmark of diseases like multiple sclerosis (MS). MS is generally considered to be an autoimmune disease, however, the causative agent of MS and a possible trigger of this disorder are still not well understood. The pathology of MS lesions is heterogeneous (Lucchinetti et al., 2000) and several patterns suggesting predominant immune-mediated inflammation (pattern I and II) and primary oligodendrogliopathy (pattern III and IV) have been described (Lucchinetti et al., 1996, 2000). Although remyelination frequently occurs after demyelinating events, it is often incomplete (Patrikios et al., 2006; Goldschmidt et al., 2009). Thus, one of the challenges of MS research is to understand remyelination failure and develop strategies to restore myelin. Animal models are a helpful tool to reveal mechanisms underlying de- and remyelination and to study the cellular response and interplay during these processes, thus providing a solid platform to elucidate putative therapeutic targets. There are four well-characterized experimental approaches to induce demyelination in the rodent CNS: genetic myelin mutations, autoimmune inflammatory-induced demyelination [experimental autoimmune encephalomyelitis (EAE)], viral-induced demyelination, genetic models, and toxic demyelination. It needs to be emphasized that all these models, including EAE, mimic only a part of MS pathology. Although EAE is probably the most commonly used model that reflects the autoimmune origin of MS, toxic demyelination is more appropriate to study remyelination. Here, cuprizone-induced intoxication has gained a lot of attention and acceptance in recent years (Matsushima and Morell, 2001; Kipp et al., 2009; Skripuletz et al., 2011a). Cuprizone [bis–cyclohexanone-oxaldihydrazone] is a copper chelating reagent, which supplemented to normal rodent chow, directly or indirectly causes oligodendroglial cell death with subsequent demyelination. Once demyelination is complete new oligodendrocytes, generated from the pool of oligodendrocyte progenitors (OPC), begin to form new myelin sheaths. Remyelination rapidly progresses after termination of the cuprizone diet (acute demyelination, Figure 1). When cuprizone is fed continuously remyelination is abortive and demyelination persists till the end of the diet (chronic demyelination). In this case the remyelination capacity retains after withdrawal from cuprizone diet but is strongly decreased (Ludwin, 1980; Lindner et al., 2009). Microgliosis and astrogliosis, driving inflammatory but also reparative processes in the face of an intact blood–brain barrier (BBB), are prominent features of this animal model (Carlton, 1966; Blakemore, 1973a; Bakker and Ludwin, 1987; Kondo et al., 1987; Hiremath et al., 1998; Matsushima and Morell, 2001; Remington et al., 2007; Gudi et al., 2009). Thus, the cuprizone model is a suitable tool to study basic glial reactions and interactions during de- and re-myelination in absence of primarily immune-mediated mechanisms.

## The cuprizone model-a historical excursion

The history of the cuprizone model spans more than 50 years. The copper chelator cuprizone was first used in clinical chemistry for quantitative analysis of copper content in biological materials such as serum and plasma (Peterson and Bollier, 1955). The attention for this compound was drawn by the findings of Carlton that cuprizone possesses various biotoxic effects in rodents like growth retardation, pregnancy disturbances, and severe CNS pathology, including demyelination, astrogliosis, and hydrocephalus (Carlton, 1966, 1967, 1969). Administration of cuprizone at dietary levels of 0.2–0.5% (w/w) in standard rodent chow to mice produced brain oedema, spongiform encephalopathy, characterized by numerous enlarged vacuoles (intracellular and intramyelinic) and creating a morphologic picture similar to that of scrapie (Suzuki and Kikkawa, 1969; Hemm et al., 1971; Kesterson and Carlton, 1971; Pattison and Jebbett, 1971a,b). In 1972 Blakemore described a massive depletion of oligodendrocytes as a primary reason for cuprizone-induced demyelination (Blakemore, 1972). He also reported about remyelination, which can occur spontaneously after removal of the cuprizone diet (Blakemore, 1973b) and established cuprizone feeding as a model to study de- and remyelination in the superior cerebellar peduncle (Blakemore, 1973a). At that time the cerebellar peduncle became the most investigated area in this animal model. In 1980 Ludwin established a chronic model of demyelination by feeding mice up to 6–7 months with cuprizone-containing chow (Ludwin, 1980). He showed that the remyelination capacity was retained but massively impaired after a long toxic insult, suggesting that the pool of remyelinating cells is limited. Similar results were reported after repeated cuprizone treatments (Johnson and Ludwin, 1981). The next 17 years only few papers concerning cuprizone were published. Moreover, most of these studies were focused on alterations of liver mitochondria (mega-mitochondria, first described by Suzuki, 1969) induced by cuprizone. In 1998 the cuprizone animal model experienced a comeback as the group of Matsushima (Hiremath et al., 1998) characterized cuprizone-induced de- and remyelination in the corpus callosum (CC) of C57BL/6 mice. In this pioneer work Hiremath et al. determined some crucial points for this model, such as age (8–10 weeks) of animals, dose [0.2% (w/w) in powdered standard rodent chow], and duration of treatment (5–6 weeks). They also described the glial reaction upon cuprizone treatment and showed that the extent of demyelination in the CC, as detected by histological staining, could be easily scored, providing investigators with a reproducible and well predictable animal model for de- and remyelination. Since then the CC is the most frequently investigated white matter tract. The use of C57BL/6 mice opened the investigators a lot of new possibilities to study genetically modified mice.

Remarkably, the susceptibility to cuprizone, extent of hydrocephalus, spatiotemporal pattern of de- and remyelination or the glial reaction are varying between species, mice strains or simply between different brain regions (Carlton, 1969; Kimberlin et al., 1976; Love, 1988; Stidworthy et al., 2003; Adamo et al., 2006; Skripuletz et al., 2010a), suggesting that the local cellular microenvironment can promote or prevent deleterious effects of cuprizone and demyelination and remyelination.

The dose of cuprizone is one of the most important factors to induce demyelination in the murine CNS. The first investigators applied a diet of 0.2–0.6% (w/w) cuprizone to different mouse strains (Carlton, 1967; Kesterson and Carlton, 1971; Ludwin, 1980), however, toxic effects such as hydrocephalus and the lethality were very high in these experiments. Hiremath et al. investigated different cuprizone dosages for C57BL/6 mice. They showed that 0.1% (w/w) cuprizone supplemented to normal rodent chow did not lead to an extensive demyelination in the CC of C57BL/6 mice, while mice fed 0.3–0.5% cuprizone displayed severe weight loss and high mortality (Hiremath et al., 1998). Thus, 0.2% (w/w) cuprizone was established to induce highly reproducible and extensive demyelination without detrimental systemic effects in 8–10 weeks old mice. However, the susceptibility to cuprizone seems to differ between young and old animals and between different mouse strains (Carlton, 1967; Blakemore, 1973b; Irvine and Blakemore, 2006). Axonal damage and glial response accompanying demyelination were more severe and prolonged in aged (6–7 months) as compared to young (8–10 weeks) mice (Irvine and Blakemore, 2006). The reason for the age-dependent susceptibility to cuprizone is currently not clear. Remyelination readily occurs in older animals but seems to be delayed or less efficient (Blakemore, 1974; Irvine and Blakemore, 2006), a finding also seen in other de-/remyelination models (Shields et al., 1999; Hinks and Franklin, 2000; Sim et al., 2002). This phenomenon may be attributed to a reduced capacity of progenitor cells to replace oligodendrocytes probably due to an altered age-related expression of certain transcription factor such as Nkx2.2 (Doucette et al., 2010) or due to an inefficient age-dependent epigenetic modulation, as shown by a declined recruitment of histone deacetylases (HDACs) to promoter regions of oligodendrocyte differentiation inhibitors (Hes1, Hes5, Id2, and Id4) and neural stem cell markers such as Sox2 followed by insufficient deacytelation of nucleosomal histones and subsequent perturbances of myelin gene expression in older mice (Shen et al., 2008).

The gender-related differences in de- and remyelination pattern were already mentioned by Ludwin who notified that Swiss female mice did not undergo demyelination (Ludwin, 1978). The demyelination pattern of SJL mice differed from that in C57BL/6 (Taylor et al., 2009). Additionally, despite of a similar extent of astro- and microgliosis as well as OPC response in both genders, female SJL mice were partially resistant to the demyelination in the CC. In contrast, the course of de- and remyelination was similar between genders in C57BL/6 mice (Taylor et al., 2010).

In the last decade it has become accepted that MS lesions arise also within gray matter structures, particularly in the cortex (Kidd et al., 1999; Bo et al., 2003). Cortical demyelination can be observed after 3 weeks of cuprizone exposure being almost complete after 6 weeks of cuprizone treatment (Skripuletz et al., 2008; Gudi et al., 2009). Furthermore, demyelination may also be detected in other brain regions such as hippocampus, basal ganglia, and cerebellum (Koutsoudaki et al., 2009; Pott et al., 2009; Skripuletz et al., 2010a).

## Mode of cuprizone action

Despite the experimental use of cuprizone for almost five decades the underlying mechanism of oligodendrocyte damage is still not completely understood. The copper-chelating property of cuprizone seems to be an obvious explanation. However, copper administration (100 ppm) synchronously to cuprizone administration (0.5%) did not reduce the toxic effects (Carlton, 1967). When mice were fed 0.2% (w/w) cuprizone and copper supplementation was increased to 130ppm the incidence of hydrocephalus declined without any effects on brain oedema and spongy degradation (Carlton, 1967). These observations pointed to a more complex mode of action that is not only based on the copper-chelating properties but may also regulate other cellular processes. Unfortunately, it is not really known whether cuprizone undergoes metabolic transformation in the organism. Extracted liver mitochondria were not affected by cuprizone *ex vivo* (Hoppel and Tandler, 1973). However, mitochondrial disturbances seem to be the key factor of oligodendroglial apoptosis, since enlarged “giant” mitochondria were detected in the liver and brain of cuprizone-treated mice (Suzuki, 1969; Hemm et al., 1971; Komoly et al., 1987). Additionally, the activities of a batch of mitochondrial enzymes containing copper as a co-factor were altered during first days/weeks of cuprizone treatment. In fact, the activity of carbonic monoamine oxidase (MAO), which is localized on the outer mitochondrial membrane, was inhibited already after 3 days on cuprizone diet and progressed to almost total inhibition in the brain until week 5 (Kesterson and Carlton, 1971; Venturini, 1973). Furthermore, the activity of cytochrome c oxidase, a terminal acceptor of the electron transport chain was as well decreased followed cuprizone administration (Venturini, 1973), while succinate dehydrogenase activity was increased in both liver and brain tissue. Within this context, Pasquini et al. reported a marked decrease in the activities of complex I, II, and III of the respiratory chain in oligodendrocyte cultures treated with cuprizone and in mitochondria isolated from cuprizone-treated mice confirming that cuprizone disturbs mitochondrial function and thus compromises the energy metabolism of cells (Pasquini et al., 2007). Recently, a strong reduction of the mitochondrial potential was also reported in cuprizone-treated oligodendrocytes *in vitro* (Benardais et al., 2013). It remains unclear why mature oligodendrocytes are preferentially vulnerable to cuprizone. A regular function of mitochondria is essential for oligodendroglial survival. Due to an extensive membrane synthesis oligodendrocytes need to display a high cellular metabolism requiring a large amount of oxygen and adenosintriphosphat (ATP). For instance, during myelination oligodendrocytes generate three times of their weight into the membrane per day and can support myelin membranes approximately 100 times the weight of their cell body (McTigue and Tripathi, 2008; Bradl and Lassmann, 2010). Furthermore, oligodendrocytes possess only a little amount of the anti-oxidative enzyme, glutathione, which also declines upon cuprizone treatment, but in comparison to other brain cells the highest amount of iron (Thorburne and Juurlink, 1996; Biancotti et al., 2008). Oligodendrocytes require iron due to intensive oxidative metabolism and as a co-factor for cholesterol and lipid biosynthesis for myelin production (Connor and Menzies, 1996; Thorburne and Juurlink, 1996). Previously, it was discussed that cuprizone may not be selective only for copper but can also chelate other heavy metals such as zinc, iron, and manganese and thus, impairs functions of additional important enzymes such as cytosolic zinc containing enzyme, anhydrase II (CA II) (Hoppel and Tandler, 1973). Besides being responsible for base-acid homeostasis, CA II is suggested to be involved in myelin metabolism and compaction since it is also activated during developmental myelination (Delaunoy et al., 1980; Ghandour et al., 1980; Komoly et al., 1987). The vast majority of the brain CA II is localized in the oligodendrocytes and the myelin (Cammer et al., 1985; Komoly et al., 1987). The activity of CA II started to decline already during the first week of cuprizone diet well before demyelination develops and (Komoly et al., 1987) persists until demyelination is complete.

In the light of all these evidences it is obvious that cuprizone directly or indirectly impairs a variety of essential cell functions. However, mitochondria seem to be mainly affected by this toxin, leading to increased production of oxidative agents, and disturbance of energy metabolism of oligodendrocytes with subsequent cell death. Additionally, since cuprizone susceptibility depends on a variety of co-factors such as age, strain, genetic background, dose, and duration of exposure, the mode of action seems to be a complex mechanism requiring multiple steps and interplayers. Inflammatory metabolites of activated microglia/astrocytes may harm oligodendrocytes as well.

## Glial reactions during cuprizone-induced de- and remyelination

### The fate of oligodendrocytes

During cuprizone-induced demyelination, oligodendrocytes start to undergo apoptosis after 3–7 days of cuprizone exposure (Mason et al., 2000a; Komoly, 2005; Hesse et al., 2010). Big vacuoles, enlarged mitochondria, and dense nuclear chromatin were observed at this time in oligodendrocytes by electron microscopy (Blakemore, 1972; Ludwin, 1978; Komoly, 2005). The mRNA expression of myelin protein genes, such as proteolipid protein (PLP), myelin-associated glycoprotein (MAG), and myelin basic protein (MBP) is dramatically down-regulated already at week 1 (Morell et al., 1998; Gudi et al., 2011). Immunohistochemically, apoptotic oligodendrocytes could be characterized by activation of caspase-3 und positive Tunel-staining. Interestingly, caspase-3-positive oligodendrocytes were detected in the CC only during the first 10 days of cuprizone diet. After 3 weeks of cuprizone diet the same amount of dying oligodendrocytes was detected in CC only via Tunel-stainig (Hesse et al., 2010), suggesting that at this time a caspase-3-independent apoptosis predominates. Veto et al. investigated apoptosis after 3 weeks of cuprizone diet and similar to Hesse et al. did not see any activation of caspase-3. Instead, they observed activation of poly(ADP-ribose) polymerase (PARP) and nuclear translocation of apoptosis-inducing factor (AIF), confirming caspase-3-independent apoptosis (Veto et al., 2010). Thus, it seems that initially oligodendrocytes are dying due to caspase-3-dependent apoptosis, while later caspase-3-independent apoptosis predominates. Similarly, enhanced PARP activation and induction of caspase-independent apoptosis were also described for pattern III MS lesions with primary oligodendrogliopathy (Veto et al., 2010). This is one of the reasons why the cuprizone model is more and more referred to as an animal model for MS pattern III (Torkildsen et al., 2008; Kipp et al., 2009; Liu et al., 2010; Veto et al., 2010; Acs and Komoly, 2012; Kang et al., 2012; Silvestroff et al., 2012). However, it always has to be kept in mind, that cuprizone-induced oligodendroglial cell death followed by myelin clearance is an artificial event directly or indirectly caused by this toxin. The oligodendroglial depletion in MS seems to progress in an apoptosis–like manner with expression of some apoptotic markers, such as bcl-2, p53, p75NTR (Dowling et al., 1996; Kuhlmann et al., 1999; Wosik et al., 2003). The transcription factor p53 is a well characterized pro-apoptotic molecule activated in response to a wide variety of toxic stimuli (Giaccia and Kastan, 1998). Li and colleagues reported that elevated levels of p53 can be detected in the CC of mice during the first 2–3 weeks of cuprizone diet. P53-deficient mice and mice systematically receiving the p53 inhibitor pifithrin are less susceptible to cuprizone-induced demyelination and show an enhanced oligodendrocytic survival (Li et al., 2008).

p75NTR is a neurotrophin receptor, which can induce different cellular responses, including cell growth but also apoptosis. p75NTR was up-regulated in the CC between day 10 and 20 of cuprizone feeding and was expressed only in oligodendrocytes. Remarkably, no simultaneous expression of p75NTR and caspase-3 could be detected (Copray et al., 2005). Since p75NTR −/− mice did not show any alteration regarding the course of de- and remyelination or glial reactions it is obvious that p75NTR does not play a major role in the induction of apoptosis in the cuprizone model.

FAS, an activator of the extrinsic apoptotic cell cascade, has been described as a key molecule for oligodendroglial cell death in EAE (Hovelmeyer et al., 2005). Upon cuprizone treatment FAS mRNA expression level was up-regulated during the first 2 weeks in the CC and thus, correlates with the expression pattern of activated caspase-3 in oligodendrocytes. However, oligodendrocyte death was not prevented in mice lacking FAS in oligodendrocytes revealing that FAS is also not essential for the induction of apoptosis in the cuprizone model (Hesse et al., 2010). After 3 weeks of cuprizone treatment mature oligodendrocytes are almost completely depleted (Hesse et al., 2010; Skripuletz et al., 2011b). From this time point onward progressing demyelination can be observed in the CC and the cortex visualized by histological and immunohistochemical techniques (Hiremath et al., 1998; Morell et al., 1998). Demyelination peaks between 4.5 and 5 weeks of cuprizone exposure in the CC and between 5 and 6 weeks in the cortex and hippocampus (Hiremath et al., 1998; Matsushima and Morell, 2001; Skripuletz et al., 2008; Gudi et al., 2009; Koutsoudaki et al., 2009). Cerebellar gray and white matter shows severe demyelination after 12 weeks of cuprizone feeding (Skripuletz et al., 2010a). It is important to mention that degradation/re-expression of different myelin proteins (at least in the CC and as depicted by immunohistochemistry) follows individual temporal pattern (Gudi et al., 2009). Complete demyelination in the medial CC can be observed by PLP staining at week 5, however, using CNPase- and MBP-antibodies already at week 4–4.5 of cuprizone feeding (Gudi et al., 2009).

Successful remyelination comprises several highly orchestrated events including migration, proliferation, terminal differentiation of OPC, and myelination (Stangel and Hartung, 2002). These repair mechanisms start very early even before demyelination can be detected immunohistochemically. Upon cuprizone-induced demyelination NG-2-positive precursor cells proliferate and accumulate in the subventricular zone (SVZ) at week 2 and then migrate towards demyelinating lesions (Mason et al., 2000a). The basic helix-loop-helix (bHLH) transcription factors Olig1 and Olig2 have been revealed to play a key role in regulating oligodendrocyte development (Zhou et al., 2000; Lu et al., 2001; Zhou and Anderson, 2002; Ligon et al., 2006). Whereas Olig2 is required for the determination of oligodendrocytes at early stages, Olig1 plays additionally a reparative role and is particularly essential for oligodendrocyte differentiation and consequent remyelination (Zhou et al., 2000; Takebayashi et al., 2002; Arnett et al., 2004). Initially identified as a downstream effector of Olig1, an oligodendrocyte specific zinc finger transcription repressor, Zfp488, was shown to interact with Olig2 physically *in vitro* and thus, being involved in the transcriptional network of Olig2 action (Wang et al., 2006). Soundarapandian et al. demonstrated that retrovirus-mediated Zfp488 over-expression could significantly promote an oligodendrogenic fate of differentiating SVZ neuronal stem cells and enhance functional recovery of cuprizone-treated mice (Soundarapandian et al., 2011).

Parallel to the myelin clearance in the CC (3–5 weeks of cuprizone administration) local and migrated OPC start to proliferate within lesions (Mason et al., 2000a; Gudi et al., 2009; Skripuletz et al., 2011b). Thereafter (up to week 5) OPC begin their terminal differentiation and restore myelin sheaths. Numerous newly generated APC/Nogo-A-positive mature oligodendrocytes can be observed from week 5.5 (0.5 weeks after the end of cuprizone feeding) in the CC (Koutsoudaki et al., 2010). On the mRNA level MBP or PLP returns to normal levels between weeks 5 and 6 (Morell et al., 1998; Gudi et al., 2011). On the protein level, newly remyelinated axons could be detected with anti-CNPase and -MBP already from week 5 and with anti-PLP and -MOG antibodies from week 5.5 to 6 (Gudi et al., 2009; Moharregh-Khiabani et al., 2010; Skripuletz et al., 2010b, 2013). Remyelination is very robust in the cuprizone model and starts directly after demyelination is complete, irrespective if cuprizone is further fed or not (Matsushima and Morell, 2001; Hiremath et al., 2008; Gudi et al., 2009). It seems that in the case of continued cuprizone feeding until week 6 newly generated oligodendrocytes are damaged again. Recently we have shown that especially differentiated oligodendrocytes but not OPC are affected by cuprizone *in vitro* (Benardais et al., 2013). Thus, in order to study remyelination in the CC it is recommended to administer cuprizone for 5 weeks to exclude any interference of beginning remyelination with cuprizone.

Upon chronic cuprizone administration (12–16 weeks) newly generated oligodendrocytes progressively undergo apoptosis. Furthermore, also the pool of OPC becomes diminished (Mason et al., 2004). Nevertheless, demyelinated axons retain the ability to be remyelinated as shown by transplantation of OPC into the lesions (Mason et al., 2004). However, the remyelination capacity was clearly reduced after the withdrawal of cuprizone (Ludwin, 1980; Lindner et al., 2009).

It is still not clear why remyelination fails in MS and a number of putative inhibitors of myelination such as LINGO-1, Notch-Jagged, and PSA-NCAM are frequently accused (Wang et al., 1998a; Charles et al., 2002; Mi et al., 2005). Antagonizing LINGO-1 in the cuprizone model enhanced differentiation of OPC and promoted remyelination (Mi et al., 2009) and remyelination was accelerated in ST8 alpha-N-Acetyl-Neuraminide alpha-2,8-Sialyltransferase 4 (St8siaIV, the polysialyltransferase responsible for PSA synthesis) knockout mice (Koutsoudaki et al., 2010), suggesting that these molecular mechanisms are indeed involved in remyelination.

### Microglia: cells with many faces

**Figure 2:**
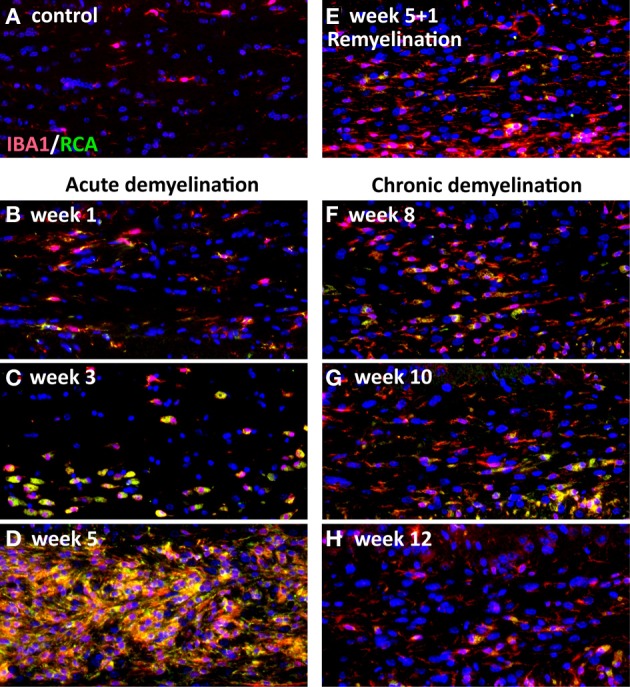
**Activation of microglia during the course of cuprizone-induced demyelination (acute and chronic).** Representative coronal sections from the medial corpus callosum were stained with the microglial marker IBA-1 (general microglial marker) and RCA-1, which stained activated microglia. There are no activated, RCA-1-positive microglia in control mice **(A)**. Activated microglia could be detected in the medial corpus callosum already after 1 week of cuprizone feeding **(B)**. After 3 weeks of cuprizone feeding activated microglia begin to accumulate in the corpus callosum **(C)**. The amount of activated microglia increases upon the course of demyelination and reaches its maximum after 5 weeks of cuprizone treatment **(D)**. If cuprizone is stopped after 5 weeks microglia numbers begin to decline with the onset of remyelination at week 6 (5 + 1 week) **(E)**. If cuprizone was fed further on to induce a chronic demyelination a small amount of activated microglia persists until week 12 **(F–H)**.

Microglia are brain-resident mononuclear phagocytes that originate from primitive macrophages in the embryonic yolk sac and colonize the neuroepithelium (in mice from day E9.5) at the onset of vascularization of the CNS (Harry and Kraft, 2012; Ginhoux et al., 2013). Microglia are active and dynamic cells, reacting very fast upon changes in the CNS (Ransohoff and Perry, 2009) microenvironment. In the acute cuprizone model activated microglia appear already at the first 2 weeks even before demyelination can be detected by histology and immunohistochemistry (Hiremath et al., 1998) (Figure 2). After 3 weeks activated microglia are numerously present in the CC, cortex, and hippocampus. In the next 2–3 weeks highly activated and proliferating microglia clear myelin debris. Once demyelination is complete the amount of activated RCA-1 (RCA, Ricinus communis agglutinin-1)/Mac-3-positive microglia starts to decrease (Mason et al., 2004; Skripuletz et al., 2011b). In contrast, activated microglia persist at a low level in chronically demyelinated lesions in mice treated with cuprizone up to 12 weeks (Mason et al., 2004; Lindner et al., 2009).

In the healthy adult brain the morphology of microglia as well as their distribution varies between different brain regions (Lawson et al., 1990). The extent of microglial accumulation during cuprizone-induced demyelination is not uniform as well. Probably primarily due to the higher amount of myelin content/debris, a significantly stronger microgliosis can be observed in the cerebellar white matter as compared to the gray matter structures (Skripuletz et al., 2008; Gudi et al., 2009). In the cerebellum microgliosis occurred delayed (from week 6 to 10), but followed a white–gray matter gradient as well (Skripuletz et al., 2010a).

In general, following stimulation with certain cytokines *in vitro* and *in vivo*, blood-derived monocytes can differentiate into two subsets of peripheral macrophages, the M1 and M2 phenotype, (Stein et al., 1992; Gordon, 2003; Edwards et al., 2006; Martinez et al., 2008; Cassetta et al., 2011). While M1 macrophages are involved in T helper cell type 1 (Th1) responses and the elimination of microorganisms, alternatively activated M2 macrophages express anti-inflammatory factors, phagocytose debris and promote tissue repair. It has been suggested that microglia can also display these two phenotypes and there is continuous debate whether microglia play a deleterious or beneficial role in CNS diseases (Schwartz et al., 2006; Block et al., 2007; Hanisch and Kettenmann, 2007; Boche et al., 2013; Chhor et al., 2013). Recent data propose that microglia can fulfill both roles and they often display an intermediate phenotype *in vivo* (Olah et al., 2012; Voss et al., 2012; Vogel et al., 2013). Conspicuously, following cuprizone-induced demyelination the peak of microglial activation tightly correlates with a massive accumulation of OPC. Similar associations are also observed in post-mortem tissue from MS patients and in EAE challenged animals (Prineas and Connell, 1979; Nishiyama et al., 1997; Wolswijk, 2002; Patani et al., 2007). A close relation between the presence of macrophages, inflammation, and OPC recruitment has been impressively confirmed by Kotter and colleagues, who demonstrated a delayed OPC recruitment after the depletion of macrophages via exposure of mice to clodronate-liposomes prior/after lysolecithin-induced demyelination. The authors linked this phenomenon to an altered growth factor expression in macrophage-depleted animals (Kotter et al., 2001, 2005).

In order to identify the microglia phenotype associated either with de- or remyelination (pro-inflammatory phenotype vs. regenerative) our group analyzed the composition of the microglial population and expression of a subset of phagocytic markers and some known pro- and anti-inflammatory cytokines in the cuprizone model (Voss et al., 2012) applying flow cytometry (FACS) technique. The most representative type of microglia during demyelination expressed CD11b^+^/CD45^low^ (CD, cluster of differentiation) and displayed a high *ex vivo* phagocytic activity accompanied by up-regulation of several phagocytic receptors such as Trem2b, FcγR II/III, CD36, and Tim-3 (Voss et al., 2012) suggesting that phagocytosis is one of the most prominent features of microglia in the cuprizone model. The expression of MHC class II was significantly increased at the maximum of demyelination (week 5) in both the CC and the cortex (Voss et al., 2012). The up-regulation of MHC-II at the peak of demyelination may be of relevance for the induction of remyelination since MHC-II −/− mice showed delayed remyelination and regeneration of oligodendrocytes after cuprizone-induced demyelination (Arnett et al., 2003). TNF-α production was strongly increased during primary demyelination in both white and gray matter microglia. In contrast, TGF-β, IFN-γ, IL-12, and IL-10 were not regulated (Voss et al., 2012). On the another hand, microglia produced growth factors such as fibroblast growth factor (FGF-2) and insulin-like growth factor I (IGF-1) that are known to be relevant for remyelination.

Expression of the T cell co-stimulatory molecule CD80 was only slightly increased on CD11b^+^/CD45^low^ microglia during de-and remyelination whereas CD40 and CD86 were not detected or only marginally expressed, suggesting that the co-stimulatory capacity of microglia in the cuprizone model was not sufficient to activate T cells. Additionally, Olah and colleges have shown that CD247 (B7-H1), a known immune inhibitory molecule (Magnus et al., 2005; Ortler et al., 2008) was up-regulated on microglia throughout de- and remyelination (Olah et al., 2012). Indeed, in response to cuprizone a very small number of T cells was recruited to the demyelinated CC and these T cells did not display an activated phenotype (Remington et al., 2007). Overall, cuprizone-induced de- and remyelination are not mediated by B or T cells, as shown by the use of RAG −/− mice that lack lymphocytes (Arnett et al., 2001; Hiremath et al., 2008). In contrast, a subset of peripheral macrophages (CD11b^+^/CD45^high^) infiltrated into the demyelinated CC of cuprizone-treated mice (McMahon et al., 2002; Remington et al., 2007). McMahon and colleagues transplanted green fluorescent protein (GFP)-positive bone marrow into irradiated C57BL/6 mice and demonstrated that macrophages constituted 1–4% of the RCA-1^+^ population at week 5–6 of cuprizone exposure (McMahon et al., 2002). Remington and co-workers reported that only 0.5% of the CD11b^+^ population is CD45^high^ and thus represents infiltrating macrophages. Our own study revealed that 5–7% of CD11b^+^/CD45^high^ cells were present in the CC at week 5 and 6, even 1 week after cuprizone diet cessation (Voss et al., 2012).

The receptors CD200R and SIRP-α, suggested to inhibit activation of microglia or keep them in a resting state (Hoek et al., 2000; Kierdorf and Prinz, 2013), were expressed on microglia at any time either in untreated or cuprizone-challenged animals. CD200R was rather up-regulated during early demyelination (Voss et al., 2012). A study from Binder et al. indicates that growth arrest gene 6 (Gas6), which is known to be a potent survival factor for a variety of cell types, could also potentially influence the activity of microglia by reducing proliferation and activation of these cells (Yagami et al., 2002; Shankar et al., 2003; Binder et al., 2008). Gas6 is acting on three tyrosine kinase receptors Tyro3, Axl, and Mer (Lai and Lemke, 1991). In untreated mice Tyro3 co-localizes with CNPase. Upon cuprizone treatment Tyro3 gene expression was down-regulated and seems to coincide with oligodendroglial loss, while Axl and Mer were found on activated microglia (Binder et al., 2008). Since Gas6-deficient mice, challenged with cuprizone, display a potentiated demyelination and microglia activation, one may hypothesize that upon cuprizone treatment activation of microglia proceeds in a tightly controlled manner by expression of an arsenal of inhibitory molecules, such as CD200R, SIRP-α, Gas6 receptors, and Trem2.

In addition, in this model microglia seem not to be assigned to a certain phenotype characteristic either for de- or remyelination since most of the genes that were regulated showed an unidirectional up-regulation or down-regulation throughout the de- and remyelination processes (Olah et al., 2012) In respect to the M1/M2 classification it seems that the microglia population in the cuprizone model is either heterogeneously composed of both M1 and M2 phenotypes or microglia display an intermediate status.

Altogether, the results suggest that in cuprizone-induced de- and remyelination activated microglia play a crucial role creating a repair promoting environment due to myelin debris clearance proceeding in a highly controlled manner, as well as by production of cytokines/growth factors essential for oligodendrocyte lineage cells.

### Astrocytes: more than glue

In the adult brain astrocytes fulfill a multitude of functions including involvement in synaptic transmission, energy metabolism, regulation of neurogenesis, maintenance of the BBB and control of cerebral blood flow (Parpura et al., 2012). Astrocytes are implicated in various CNS disorders, e.g., Alzheimer's disease (Parpura et al., 2012) and demyelinating disorders such as neuromyelitis optica and MS (Nair et al., 2008; Kipp et al., 2011a). Astrocytes in MS are considered to possess a dual role since beneficial as well as detrimental effects of astrocytes on de- and remyelination are being discussed (Williams et al., 2007; Nair et al., 2008; Kipp et al., 2011a; Moore et al., 2011). In response to different CNS pathologies astrocytes become activated whereupon GFAP-expression is increased and hypertrophy of cellular processes occurs, a process called astrogliosis (Pekny and Nilsson, 2005).

Already in the 1960s Carlton reported that cuprizone ingestion in mice led to enlargement and hyperplasia of astroglia in brain regions that showed signs of cuprizone-induced demyelination and that astrocytosis preceded loss of myelin (Carlton, 1967). Strong astrogliosis comprising hypertrophy and hyperplasia of astrocytes occurs as a response to cuprizone treatment in different white and gray matter structures for instance the CC (Matsushima and Morell, 2001; Gudi et al., 2009), hippocampus, (Koutsoudaki et al., 2009; Norkute et al., 2009), cerebellum, cortex (Skripuletz et al., 2008), and basal ganglia (Pott et al., 2009). Since the CC is the most-investigated brain-region in the cuprizone mouse model, the chronology and peculiarity of astrocytosis will shortly be explained in this white-matter area. Astrocytes in control animals show few, delicate and short processes, whereas already during the first 2 weeks of cuprizone exposure astrocytes display morphological changes and become hypertophic with thick processes. The number of astrocytes is not significantly increased until 3 weeks of cuprizone treatment, simultaneously with first signs of apparent demyelination. Concomitantly, astrocytes display signs of proliferation during the course of acute demyelination (Hiremath et al., 1998; Gudi et al., 2009). Correlating with the degree of demyelination, astrogliosis is increasing during acute demyelination peaking around the time point of maximal demyelination and incipient remyelination around 5–6 weeks of cuprizone exposure (Hiremath et al., 1998; Gudi et al., 2009). Under continuing cuprizone treatment astrogliosis persists and after chronic demyelination the number of astrocytes is similar to acute demyelination (Lindner et al., 2009; Kipp et al., 2011b). Since astrocytes show significantly lower gene and protein expression of GFAP after chronic demyelination compared to acute demyelination, the activation status of astrocytes appears to be higher after acute demyelination (Ludwin, 1980; Kipp et al., 2011b). After cessation of cuprizone treatment after acute and chronic demyelination astrogliosis is persisting over a period of weeks during the remyelination period, which is in contrast to the transient activation of microglia (Skripuletz et al., 2011a; Hibbits et al., 2012). Astrogliosis is related to glial scar formation particularly in chronic stages of MS, however, in chronic cuprizone lesions there are no signs of astroglial scar properties (Hibbits et al., 2012).

It is well known that astrocytes produce different growth factors during cuprizone-induced demyelination, thereby promoting OPC proliferation by FGF-2 and platelet-derived growth factor alpha (PDGFα), or regulating oligodendrocyte differentiation and survival via ciliary neurotrophic factor (CNTF). Recently, the expression of the radial glia cell marker fatty acid binding protein 7 (FABP7) was found to be induced in activated astrocytes after cuprizone-induced acute demyelination whereas expression of this marker and the extent of astrogliosis was diminished after chronic demyelination (Kipp et al., 2011a). Interestingly, the expression of FABP7 in astrocytes also correlated with remyelination of lesions in EAE and MS and induced the expression of various growth factors *in vitro* [FGF2, PDGFα, and osteopontin (OPN)] indicating a potential beneficial role of FABP7 expression in activated astrocytes for remyelination.

In a recent study from our lab, the role of astrocytes in experimental demyelination was investigated by loss-of-function experiments in which astrocytes where depleted in mice treated with cuprizone (Skripuletz et al., 2013). Ablation of astrocytes during cuprizone-induced demyelination had no impact on the loss of mature oligodendrocytes and on axonal damage, but resulted in a diminished activation and invasion of microglia into the demyelinated lesion. As a consequence the removal of myelin debris was delayed and subsequent OPC proliferation and remyelination were delayed. It was concluded, that astrocytes are responsible for the recruitment of microglia. In addition, the depletion of astrocytes resulted in diminished numbers of mature oligodendrocytes during remyelination (Skripuletz et al., 2013).

A close relation between the presence of astrocytes and the efficiency of remyelination has already been recognized for a long time (Ludwin, 1980; Blakemore, 1981). However, the exact role of astrocytes in de- and remyelination is still not fully understood. There is increasing evidence that astrocytes are key players in complex interactions with OPC, mature oligodendrocytes, and microglia supporting remyelination at least in the cuprizone model.

## Crosstalk between glia

### Inflammation-related cytokines and molecules

In the cuprizone model severe microglial accumulation and astrogliosis are promoting an intensive inflammatory response (Blakemore, 1972, 1973a; Hiremath et al., 1998) associated with the expression of a battery of inflammatory cytokines and chemokines including pro-inflammatory molecules such as TNF-α, interleukin-1β (Il-1β), interferon- γ (IFN-γ), and nitric oxide (NO) (Hiremath et al., 1998; Arnett et al., 2001; Mason et al., 2001; Voss et al., 2012). A number of *in vitro* studies revealed that these molecules can irreversibly harm oligodendrocytes (Selmaj and Raine, 1988; Merrill et al., 1993; Vartanian et al., 1995; Baerwald and Popko, 1998). However, a growing body of evidence corroborates the assumption that an adequate and controlled inflammatory response is required for a successful myelin regeneration promoting the clearance of myelin debris and stimulating proliferation/regeneration of myelin-forming oligodendrocytes (Diemel et al., 1998; Robinson and Miller, 1999; Arnett et al., 2001; Kotter et al., 2001, 2005, 2006; Setzu et al., 2006; Biancotti et al., 2008; Merson et al., 2010).

Matrix metalloproteinases (MMP), a family of zinc-dependent endopeptidases, are highly involved in the perpetuation of inflammation, myelin degradation, and remodeling of extracellular matrix but also proposed to modulate different physiological processes such as cellular development/growth, survival, apoptosis, and migration (McCawley and Matrisian, 2001; Malemud, 2006). Furthermore, MMP are shown to be implicated in numerous regenerative processes (Larsen and Yong, 2004; Yong, 2005; Lehmann et al., 2009). Previously, our group could show significant changes in the expression of several MMP, especially during the remyelination phase, arguing for a regulatory role of MMP in tissue regeneration (Skuljec et al., 2011). In particular, the expression of MMP11, MMP12, and MMP3 was clearly increased during remyelination. MMP12 was additionally up-regulated during the demyelination phase, however, MMP7, -9, -11, -13, -15 were either not regulated or even down-regulated during myelin clearance in the CC. In contrast, the expression of endogenous antagonists of metalloproteinases (TIMP) was increased during the demyelination peak between weeks 4.5 and 5. TIMP2 was found to be expressed by microglia (Skuljec et al., 2011), whereas the source of TIMP1 in the CNS is linked to astrocytes (Gardner and Ghorpade, 2003). MMP3 was localized exclusively in activated astrocytes, whereas MMP12 appeared in microglia, astrocytes and during the remyelination phase in oligodendrocytes as well. The balance between MMP and TIMP is a very critical factor for the development/perpetuation of different pathologies (Kieseier et al., 1999; Lindberg et al., 2001). It seems, however, that in the cuprizone model deleterious effects of MMP and inflammatory processes themselves are tightly controlled by TIMP. Interestingly, the expression of MMP can be regulated by some inflammatory cytokines, such as IL-1β or TNF-α (Dasilva and Yong, 2008).

TNF-α is a multipotent inflammatory cytokine, which can act through two receptors, TNFR1(p55) and TNFR2(p75) (Vandenabeele et al., 1995; Locksley et al., 2001), triggering multiple and sometimes opposing effects (apoptosis vs. proliferation). Only TNFR1(p55) possesses a cytoplasmic protein interaction region called “death domain” and may directly induce apoptosis via activation of different caspases. However, the signaling through TNFR1(p55) is complex and can also trigger a nuclear translocation of NF-kappa B and transcription of its target genes including both anti-apoptotic factors (e.g., IAPs, Bcl-2, Bcl-xL) and pro-inflammatory factors (e.g., cytokines, chemokines) (Figiel, 2008). TNFR2(p75) responds preferentially to membrane-bound TNF-α and does not contain a death domain (Grell, 1995). In EAE, TNFR1(p55) is supposed to be responsible for the detrimental effects of TNF-α by initiating and exacerbating the course of disease while immunosuppressive and immunoregulatory properties of TNF-α are thought to be mediated by action through TNFR2(p75) (Eugster et al., 1999; Suvannavejh et al., 2000; Kassiotis and Kollias, 2001). In MS, clinical therapies aimed to block TNF-α-mediated pro-inflammatory effects (Van Oosten et al., 1996; The Lenercept Multiple Sclerosis Study Group and The University of British Columbia MS/MRI Analysis Group, 1999; Sicotte and Voskuhl, 2001) did not yield in an amelioration of disease and even worsened the clinical course in some patients, strongly suggesting a bivalent role of TNF-α in demyelination and myelinogenesis. In the cuprizone model, TNF-α was demonstrated to act as well in a bivalent manner since TNF-α −/− mice displayed a reduced loss of oligodendrocytes during the first 3.5 weeks of cuprizone feeding but also a significantly diminished ability to remyelinate (Arnett et al., 2001). In this study TNFR2 but not TNFR1 has been shown to be gradually up-regulated during de- and remyelination (Arnett et al., 2001). Furthermore, similar to TNF-α-deficient mice TNFR2 −/− mice displayed diminished/delayed regeneration of myelin suggesting that beneficial effects of TNF-α on proliferation of OPC and subsequent remyelination are promoted through the TNFR2 pathway (Arnett et al., 2001). Interestingly, depletion of TNF-α did not alter the extent of microglial or astroglial responses, however, it displayed significant effects on expression of inflammatory genes. In particular, several genes of the MHC-family were reduced in the mice lacking TNF-α (Arnett et al., 2003). As already mentioned, MHC-II −/− mice showed a delayed remyelination and regeneration of oligodendrocytes after cuprizone-induced demyelination (Arnett et al., 2003), again emphasizing a beneficial role of the inflammatory response in the repair processes after a demyelinating insult.

Lymphotoxin-α (Lt-α), or TNF-β, belongs to the TNF-superfamily and acts through TNFR1, TNFR2, and lymphotoxin-β receptor (LtβR). In the cuprizone model, Lt-α is expressed primarily by astroglia (Plant et al., 2005) while LtβR by microglia (Plant et al., 2007). Mice lacking Lt-α showed a decreased number of microglia and delayed demyelination as well as reduced loss of mature oligodendrocytes at week 3.5 (Plant et al., 2005). The course of remyelination was not influenced by this mutation. However, remyelination was slightly delayed in mice lacking LtβR (Plant et al., 2007). Surprisingly, remyelination was enhanced when LtβR-signaling was inhibited by an LtβR-Ig fusion decoy protein. These controversial results were associated with developmental abnormalities in the immune system of LtβR lymphotoxin-β receptor −/− mice (Plant et al., 2007).

IL-1β is another prominent pro-inflammatory cytokine that is produced primarily by activated microglia and macrophages (Giulian et al., 1986; Sairanen et al., 1997). IL-1β induced proliferation of astrocytes (Giulian and Lachman, 1985; Giulian et al., 1988) and activated the production/release of different cytokines such as TNF-α, interleukin-6 (IL-6), NO and thus contributes to the inflammatory response. On the other hand, IL-1β increased the secretion of growth factors promoting proliferation/differentiation of OPC (Lee et al., 1995; Rothwell and Luheshi, 2000; Vela et al., 2002; Allan et al., 2005). In the cuprizone model IL-1β was gradually up-regulated from the first week of cuprizone intoxication, then strongly increased at week 3 and sustained on this level until week 6 (Mason et al., 2001). The cellular sources of IL-1β were Mac-1-positive microglia and a subpopulation of astrocytes. In this study Mason and colleagues have shown that differentiation of OPC and thus, remyelination are dramatically reduced in absence of IL-1β probably due to IL-1β-dependent reduction of IGF-1 production, which is known to promote differentiation of OPC and myelination (Mozell and Mcmorris, 1991). Interestingly, accumulation of microglia and proliferation of OPC were not altered in the IL-1β −/− mice. Additionally, IL-1β did not display any impact on depletion of oligodendrocytes following cuprizone treatment (Mason et al., 2001).

The matricellular phosphorylated glycoprotein osteopontin (OPN) is highly expressed in bone but is also secreted by a variety of cell types including endothelial cells, activated macrophages, leukocytes, and T cells (Murry et al., 1994; Ashkar et al., 2000; Kury et al., 2005). OPN is known to possess multiple biological and immunoregulatory functions being involved in cell adhesion and providing a chemotactic stimulus for macrophages and astrocytes (Denhardt and Guo, 1993; Denhardt and Noda, 1998; Giachelli et al., 1998; Wang et al., 1998b; Sodek et al., 2000; Mazzali et al., 2002). However, the precise role of this molecule in the CNS is not completely understood. Previously, OPN was shown to be up-regulated in MS lesions and in several experimental animal models for demyelination including the cuprizone model (Chabas et al., 2001; Selvaraju et al., 2004; Zhao et al., 2008). Upon cuprizone treatment the expression of OPN was increased on the peak of demyelination and during early remyelination in activated astrocytes and microglia. Additionally, OPN was reported to enhance the proliferation capacity of OPC cell lines and promote both MBP synthesis and myelin formation in mixed cortical cultures, suggesting to be involved in remyelination (Selvaraju et al., 2004).

NO is a highly reactive molecule mainly released by activated immune cells including microglia during different inflammatory processes. An overproduction of NO is known to induce an irreversible cell damage and death in different cell populations (Chao et al., 1992; Merrill et al., 1993). However, the role of NO in the CNS seems to be controversial. Although in EAE the expression level of inducible NO synthase (iNOS), an enzyme producing NO, correlates with severity of disease and cellular infiltrate (Okuda et al., 1995; Cross et al., 1996; Tran et al., 1997) some studies have shown even a protective role of NO. In these studies the lack of iNOS leads to an earlier onset and more severe disease course of EAE (Fenyk-Melody et al., 1998; Sahrbacher et al., 1998). Similar in the cuprizone model, a depletion of iNOS exacerbated demyelination (Arnett et al., 2002), suggesting that its role may also include the regulation of some regenerative processes. On the other hand, the absence of constitutively expressed neuronal NO synthase (nNOS) protected the mice from cuprizone-induced demyelination, reduced the inflammatory glial response and oligodendroglial apoptosis, but also impaired OPC accumulation and subsequent remyelination (Linares et al., 2006). The expression of TNF-α, Il-1β, and IGF-1 was significantly decreased in nNOS −/− mice underlining the dual role of inflammation on de- and remyelination.

Similar contradictory observations were reported about the impact of IFN-γ on de- and remyelination in the cuprizone model. First Gao et al. proposed protective effects of IFN-γ on myelin using mice that ectopically expressed a low level of IFN-γ under the MBP promoter in oligodendrocytes (Gao et al., 2000). In these mice reduced loss of oligodendrocytes following cuprizone intoxication was associated with an increase of IGF-1 expression, which is known to be protective against cytokine-mediated oligodendroglial cell death (Ye and D'Ercole, 1999; Mason et al., 2000b). Later, Mana et al. reported a deleterious role of IFN-γ demonstrating reduced demyelination and enhanced remyelination in mice lacking the binding chain of the IFN-γ receptor (Mana et al., 2006). In both cases, however, microglia were suggested to be regulated by IFN-γ, since MBP/IFN-γ transgenic animals displayed slightly elevated numbers of GFAP-positive and RCA-1-positive glial cells already prior to cuprizone administration (Gao et al., 2000). In contrast, in IFN-γR −/− mice cuprizone-induced microglia accumulation was initially diminished or simply delayed (Mana et al., 2006) that was reflected at week 3 of cuprizone diet by a reduced expression of microglia-associated molecules such as IGF-1 and TNF-α that have been proposed to promote proliferation of OPC. Nevertheless, Mana et al. reported an increased number of NG2-positive cells in IFN-γR −/− mice in comparison to wild type (wt) mice suggesting inhibitory/harmful effects of IFN-γ on OPC recruitment/survival and remyelination (Mana et al., 2006). In parallel, the group of Popko reported in transgenic mice that ectopically expressed IFN-γ under the GFAP promoter in a tetracycline-controlled manner an altered but not inhibited recruitment/expansion of OPC via IFN-γ-signaling. Nevertheless, the remyelination capacity was significantly improved in mice lacking IFN-γ, probably linked to reduced endoplasmic reticulum stress via IFN-γ (Lin et al., 2006). In this study, a deleterious effect of IFN-γ on remyelination was also confirmed in EAE (Lin et al., 2006). In conclusion, these conflicting results may allow the assumption that the effects of IFN-γ and other pro-inflammatory molecules, whether harmful or helpful, are dependent on their quantitative and temporal expression pattern.

### Growth factors

Growth factors are known to possess pleiotropic effects in a variety of cell types including neuronal and glial cells in the CNS. Together with chemokines, cytokines, and hormones growth factors are strongly involved in the orchestration of development, specification, and maintenance of CNS structures. Growth factors modulate plasticity of the CNS and its repair, including generation of new glial and neuronal cells, their migration, proliferation, maturation, and myelination. A disturbed balance of interacting growth factors that regulate differentiation of oligodendrocytes and onset of myelination may contribute to the limited remyelination of MS plaques (Franklin and Hinks, 1999; Miller and Mi, 2007). Gaining a clearer understanding of the role of growth factors in de- and remyelination we assessed a temporally detailed mRNA expression profile of different growth factors in the cuprizone model (Gudi et al., 2011). We found that several growth factors such as FGF-2, CNTF, IGF-1, and glial cell-derived neurotrophic factor (GDNF) are strongly regulated during cuprizone treatment and/or in the recovery phase.

It is widely accepted that the main impact of FGF-2 on OPC is the induction/support of proliferation (Bansal et al., 1996), inhibition of progenitor differentiation, and subsequent myelination (Bansal and Pfeiffer, 1997; Goddard et al., 2001; Murtie et al., 2005a,b; Zhou et al., 2006). In our own studies we found that the FGF-2 mRNA expression began to increase already from the first weeks of cuprizone feeding and was even stronger up-regulated after 4 weeks (severe demyelination) corresponding to the intensive proliferation of OPC (Gudi et al., 2011). Taking advantage of FGF-2 −/− mice Armstrong et al. have shown that the absence of FGF-2 leads to a faster regeneration/differentiation of oligodendrocytes after cuprizone-induced demyelination (Armstrong et al., 2002). However, an up-regulation of FGF-2 mRNA expression during remyelination phase has been reported in lysolecithin-induced demyelination (Hinks and Franklin, 1999) as well as *in vitro* in myelinating aggregate cultures (Copelman et al., 2000). Thus, it can be suggested that FGF-2 possesses multiple functions and acts not only directly on oligodendrocytes but also influences other cell types promoting myelination indirectly. Furthermore, FGF-2-signaling is tightly regulated by binding to different FGF receptors and their splice variants resulting in different modes of actions (Bansal et al., 1996, 2003; Reuss and Von Bohlen Und Halbach, 2003; Eswarakumar et al., 2005; Fortin et al., 2005). A recent study of Zhou and colleagues showed that all four FGF receptors are highly regulated upon cuprizone treatment and during remyelination (Zhou et al., 2012). In this study the authors pursued a genetic approach with mice lacking FGFR1 in oligodendrocytes in a tamoxifen-dependent manner that were treated for 12 weeks with cuprizone. Previously, FGFR1 was shown to be involved in OPC proliferation as well as in the inhibition of OPC differentiation *in vitro* (Bansal et al., 1996; Zhou et al., 2006). In Zhou et al. study after a recovery period for additional 6 weeks, OPC numbers were diminished and the number of mature oligodendrocytes was increased, indicating a beneficial effect of FGFR1 reduction on OPC differentiation (Zhou et al., 2012). Additionally, an inhibitory effect of FGF-2-signaling on OPC differentiation was observed in FGF −/− mice upon chronic demyelination. Interestingly, FGF-2 level persisted to be elevated until 12 weeks of cuprizone exposure, failing to potentiate proliferation of OPC significantly, since both the wt mice and FGF −/− mice displayed a similar number of OPC (Armstrong et al., 2006), suggesting that FGF-2 may not be an essential/exclusive mitogenic factor for OPC *in vivo*, and may even predominantly promote an inhibitory effect impeding OPC differentiation and remyelination (Armstrong et al., 2006). Although these studies did not provide detailed insights into the responses of other glial cells, we believe that FGF-2 modulates not solely the oligodendrocyte lineage but may also influence the action/interaction of astrocytes and microglia. FGF-2 as well as FGFRs can be expressed by both activated astrocytes and microglia while oligodendroglial cells expressed mainly FGFRs (Bansal et al., 1996; Messersmith et al., 2000; Gudi et al., 2011).

The second growth factor which has been intensively studied in the cuprizone model is platelet-derived growth factor α (PDGFα). Being a potent mitogen for OPC PDGFα is one of the prominent compounds of the culture medium of primary cortical OPC derived from new-borns or embryos as well as immortalized OPC *in vitro* (Noble et al., 1988; Bansal et al., 1996). The PDGFα receptor (PDGFαR) is widely accepted as a phenotypic marker for this developmental stage of the oligodendrocytes lineage (Hart et al., 1989). Astrocytes were shown to produce PDGFα/PDGFβ (Pringle et al., 1989) stimulating OPC to proliferate/differentiate *in vitro* (Raff et al., 1988). In the developing murine CNS PDGF was proposed to determine the OPC population size (Calver et al., 1998; Van Heyningen et al., 2001). To investigate the impact of PDGFα on de- and remyelination Woodruff and colleagues applied cuprizone or lysolecithin to mice over-expressing human PDGFα under the GFAP promoter in astrocytes. These transgenic mice displayed an increased amount of OPC in steady state conditions. After a demyelinating insult due to either cuprizone or lysolecithin the OPC density increased highly in hPDGFα-GFAP mice as compared to wt mice. Since there was no significant enhancement of remyelination in the lysolecithin model the authors concluded, that the OPC density seems not to be a rate-limiting factor for a successful remyelination (Woodruff et al., 2004). Another study showed an impaired oligodendrocyte repopulation in PDGFαR heterozygous knockout mice, caused by a reduced proliferation capacity and overall density of OPC arguing for an important role of PDGFαR-signaling on OPC proliferation. Differentiation of OPC was, however, not altered in PDGFαR +/− mice (Murtie et al., 2005b). Later, the same group reported an enhanced remyelination, increased regeneration, and reduced apoptosis of oligodendrocytes in the hPDGFα-GFAP mice (exploited also by Woodruff et al., 2004) after chronic cuprizone intoxication (Vana et al., 2007). Nevertheless, it remains unclear how PDGFα-signaling impacts microgliosis and astrogliosis in these transgenic mice (hPDGFα-GFAP, PDGFαR +/−), since it is known that astrocytes also express PDGFαR and respond to PDGFα (Besnard et al., 1987; Fruttiger et al., 1996).

IGF-1 is believed to play a crucial role in oligodendrocyte differentiation, survival, and myelination (Mozell and Mcmorris, 1991; Barres et al., 1992, 1993; Goddard et al., 1999; Ye and D'Ercole, 1999). IGF-1 over-expressing mice showed hypermyelinated axons (Ye et al., 1995), whereas IGF-1 knockout mice exhibited a decreased number of oligodendrocytes and hypomyelination (Beck et al., 1995; Ye et al., 2002). The involvement of IGF-1 in de- and remyelination has been reported for various experimental demyelination models (Yao et al., 1995; Hinks and Franklin, 1999; Mason et al., 2000b, 2003). In the CC of cuprizone-treated mice the expression of the IGF-1 mRNA started to be up-regulated after 2 weeks of cuprizone feeding (Gudi et al., 2011) and peaked at weeks 4–5 corresponding to the maximal demyelination, proliferation of OPC and onset of their differentiation (Gudi et al., 2011). Komoly et al. investigated de- and remyelination in the cerebellar peduncles in swiss Webster mice exposed to 0.6% (w/w) cuprizone and found an astrocytic expression of IGF-1, whereas IGF-1 receptor (IGF1R) was transiently expressed in OPC (Komoly et al., 1992) during the early recovery phase. In our own studies we could confirm this finding; however, additionally we observed IGF-1 mRNA expression in activated microglia (Voss et al., 2012). Interestingly, microgliosis was more pronounced in mice that constantly over-expressed IGF-1 within the brain after 3 weeks of cuprizone diet (Mason et al., 2000b). Additionally, these mice exhibited an enhanced demyelination at week 3 but an almost complete myelin recovery at week 5 in contrast to wt mice undergoing a severe myelin deficit at this time point. Furthermore, instead of being nearly completely depleted, mature oligodendrocytes were numerously present in the CC of IGF-1 over-expressing mice during demyelination suggesting a highly protective role of IGF-1 on oligodendrocytes. The positive effect of IGF-1 on mature oligodendrocyte survival was also observed *in vitro* (Barres et al., 1992, 1993; Ye and D'Ercole, 1999). Moreover, these surviving oligodendrocytes even seem to retain the ability to remyelinate timely on week 5, regardless of remaining myelin debris (Mason et al., 2000b). In conditional mouse mutants, in which the expression of the *Igf1r* gene was ablated exclusively in neurons and OPC of the cerebrum, there was a reduced accumulation of OPC, leading to a decreased amount of newly generated oligodendrocytes (Mason et al., 2003).

CNTF and leukemia inhibitory factor (LIF) are acting via heterodimeric gp130/LIFRβ (CNTF requires additionally CNTFRα) receptor complex. Both molecules possess multiple functions in a variety of cell types including promotion of oligodendrogenesis, differentiation, myelin synthesis, and survival of oligodendrocytes (Mayer et al., 1994; Barres et al., 1996; Marmur et al., 1998; Stankoff et al., 2002; Talbott et al., 2007; Ishibashi et al., 2009) *in vitro* and *in vivo* and can prevent oligodendrocyte death under pro-inflammatory conditions *in vitro* (Louis et al., 1993; Vartanian et al., 1995). In cuprizone-induced demyelination LIF-deficient mice displayed more severe demyelination, increased loss of oligodendrocytes, and an impaired remyelination (Emery et al., 2006; Marriott et al., 2008). Remarkably, the oligodendrocyte replenishment, which includes both OPC proliferation and differentiation, seemed not to be significantly compromised in these transgenic mice, arguing for a direct effect of LIF on differentiated oligodendrocyte survival and myelination (Marriott et al., 2008). Exogenously administered LIF limited cuprizone-induced demyelination in wt mice and rescued the LIF-knockout phenotype during cuprizone-induced demyelination. However, remyelination was not further enhanced if wt mice were complemented with LIF during the recovery phase (Marriott et al., 2008). In our own study we were able to show that LIF mRNA expression was up-regulated very early mainly during the first 3 weeks of cuprizone administration in both CC and cortex thereafter decreasing to normal levels in the recovery phase after toxin withdrawal (Gudi et al., 2011). Since LIF was proposed to be chemotactic and mitogenic for macrophages (Sugiura et al., 2000; Kerr and Patterson, 2004) and able to stimulate myelin uptake *in vitro* (Hendriks et al., 2008) it can be speculated that also in cuprizone-induced demyelination LIF regulates microglia attraction and activation. Interestingly, LIF can inhibit the production of the pro-inflammatory cytokine TNF-α and reactive oxygen species (ROS) by macrophages probably limiting a progression of inflammation and acting anti-apoptotic (Hendriks et al., 2008).

The results of the study by Sabo et al. indicated an important role for bone morphogenic protein (BMP) in modulating oligodendrogliogenesis and remyelination (Sabo et al., 2011). Several BMPs including BMP4 belong to the transforming growth factor-superfamily (TGF) of cytokines. TGF-β1 mRNA expression itself was significantly enhanced during cuprizone ingestion showing the same temporal pattern as IGF-1 (Gudi et al., 2011). OPC and mature oligodendrocytes express BMP4 and its receptors (Kondo and Raff, 2004). *In vitro* treatment of OPC with BMP4 inhibited their differentiation but promoted differentiation of astrocytes (Mabie et al., 1997; Grinspan et al., 2000). *In vivo*, the expression of BMP4 was elevated in OPC within demyelinated lesions induced by ethidium bromide (Zhao et al., 2005). Similarly, in EAE BMP4 was up-regulated upon demyelination (Ara et al., 2008), suggesting an important role for BMP-signaling in demyelinating events. The BMP-pathway was also activated in astrocytes and Olig2-positive cells during cuprizone-induced demyelination (Sabo et al., 2011). An intraventricular delivery of BMP4 for 7–14 days (4–5 or 4–6 week of cuprizone diet) transiently increased the proliferation predominantly of OPC and to a lower degree of astrocytes and microglia. However, this augmented pool of OPC failed to differentiate into mature oligodendrocytes and to enhance remyelination, probably due to induction of apoptosis in these cells by exogenously administered BMP4. Conversely, infusion of Noggin, an antagonist of the BMP4-pathway, promoted maturation of oligodendrocytes and potentiated remyelination in the CC after cuprizone withdrawal (Sabo et al., 2011). However, the sequential delivery of BMP4 between weeks 4 and 5 followed by Noggin infusion for the following week did not improve remyelination (Sabo et al., 2013), indicating the complexity of growth factor fine tuning and interplay.

### Chemokines

Chemokines were primarily postulated to regulate directional migration of leukocytes. However, it becomes more and more evident that different chemokines are also implicated in a variety of fundamental biological functions including proliferation, migration, and differentiation of different cell types playing a crucial role in development, homeostasis, and plasticity of the immune system and the CNS (Zou et al., 1998; Bajetto et al., 2002; Rezaie et al., 2002; Tran and Miller, 2003; Le et al., 2004; Tran et al., 2004; Raman et al., 2011). Upon cuprizone-induced demyelination mRNA expression of chemokine CCL3 was initiated already after 2 days and then gradually increased until week 4 (McMahon et al., 2001; Buschmann et al., 2012). The deficiency of this chemokine resulted in the reduced recruitment of microglia and astrocytes as reflected by delayed demyelination and an attenuated TNF-α secretion after 3.5 weeks of cuprizone ingestion (McMahon et al., 2001). CCL3 has been reported to be produced by lipopolysaccharide (LPS)-stimulated microglia and is implicated in the migration and activation of these glial cells *in vitro* and *in vivo* (Hayashi et al., 1995; Cross and Woodroofe, 1999; Selenica et al., 2013). Production of CCL3 in murine cortical astrocytes was also regulated after an inflammatory stimulus such as LPS (Murphy et al., 1995).

CCL2 is another well characterized chemokine known to be strongly implicated in the pathology of MS and EAE. In cuprizone-induced demyelination CCL2 expression was a transient phenomenon (McMahon et al., 2001; Buschmann et al., 2012) being significantly up-regulated particularly during the first week of cuprizone exposure. CCL2 has been shown to be produced by astrocytes in EAE (Ransohoff et al., 1993; Adamus et al., 1997). LPS, IL-1β, and TNF-α-induced CCL2 secretion by astrocytes, but not microglia *in vitro* (Hayashi et al., 1995). Additionally, this study showed that CCL2 induces chemotaxis of microglia. EAE studies using neutralizing antibodies against CCL2 and CCL3 suggested that CCL3 controls accumulation of mononuclear cell during acute EAE, correlating with increasing acute disease severity, while CCL2 was responsible for a mononuclear cell infiltration during relapsing EAE (Karpus et al., 1995; Kennedy et al., 1998). Thus, it might be proposed that in the cuprizone model CCL2 modulates an early attraction or activation of microglia to the lesion site, while CCL3 plays an important role in later stages, possibly promoting proliferation or initiation of phagocytosis of myelin debris. In line with other studies (McMahon et al., 2001; Buschmann et al., 2012) we could also detect an up-regulated expression of CCL2 and CCL3 upon cuprizone treatment (Skripuletz et al., 2013). Interestingly, a partial ablation of astrocytes in mice challenged for 3 weeks with cuprizone did not reveal any difference in CCL2 and CCL3 expression, suggesting that these cytokines are not astrocyte-derived at least at this time point (Skripuletz et al., 2013). However, the clearance of damaged myelin as well as the recruitment of microglia was strongly impaired in this experimental setting, arguing for astrocyte-derived factors that mediate attraction of microglia. Our studies suggested that this process may be regulated by the chemokine CXCL10 since CXCL10 mRNA expression was reduced in the astrocyte-depleted mice and the treatment of astrocyte cultures with LPS, IL-1β, or TNF-α led to an enhancement of CXCL10 production *in vitro* (Skripuletz et al., 2013).

The chemokine CXCL12 is a potent chemoattractant for lymphocytes and monocytes but not neutrophils (Bleul et al., 1996). CXCL12–CXCR4-signaling plays an important role in the patterning and plasticity of both immune and nervous system during development and adulthood (Klein and Rubin, 2004; Li and Ransohoff, 2008). In the adult CNS, CXCL12 orchestrates neurotransmission, neurotoxicity and neuroglial interactions (Lazarini et al., 2003). Activation of the CXCR4 cascade in OPC leads to migration, proliferation, and differentiation of these cells (Dziembowska et al., 2005; Kadi et al., 2006; Maysami et al., 2006). The cellular source of CXCL12 within the CC of cuprizone-treated mice includes both GFAP-positive astrocytes and CD11b-positive microglia. CXCR4 was located on NG-2-positive OPC. Loss of CXCR4-signaling via either pharmacological blockade or *in vivo* RNA-silencing led to reduced OPC maturation and remyelination failure in the cuprizone model (Patel et al., 2010). Astrocytic expression of CXCL12 was detected in MS lesions and could be as well induced upon IL-1β, TNF-α, and MBP stimulation *in vitro* (Ambrosini et al., 2005; Calderon et al., 2006). Activated astrocytes are armed with both TNFR1 and TNFR2. Interestingly, mice lacking TNFR2 displayed a loss of CXCL12 up-regulation in astrocytes and concomitant reduction in the number of NG2- CXCR4-positive OPC leading to delayed remyelination in the cuprizone model (Patel et al., 2012). The lentiviral-gene delivery of CXCL12 into TNFR2 −/− mice could rescue this effect, suggesting a TNF-α-mediated mechanism of remyelination via astrocytic CXCL12 (Patel et al., 2012).

## Is the cuprizone model relevant for MS research? limitation and strength of different animal models

Since dysfunctions of the immune system are supposed to be the main compound in the pathology of MS, most currently available MS therapies focus on the suppression or control of immune-mediated mechanisms. However, the aetiology of MS remains still unresolved and the pathology of MS lesions as well as the individual course of disease are highly heterogeneous (Lucchinetti et al., 2000; Lassmann et al., 2007). Moreover, known immunosuppressive therapeutics that are quite effective in relapsing-remitting MS (RRMS) extend limited beneficial effects during late stages of secondary-progressive (SPMS) and primary-progressive (PPMS) MS subtypes (Bradl and Lassmann, 2009). Therefore, the focus in MS research is directed towards the development of individual MS therapies and the establishment of new regenerative agents that support repair mechanisms (Lucchinetti et al., 1997; Stangel and Hartung, 2002; Huang et al., 2011; Zhang et al., 2011; Hagemeier et al., 2012; Franklin and Gallo, 2014). Probably the most promising approach for MS treatment would include an immunomodulatory, protective, and regenerative compound (Zhornitsky et al., 2013). A broad spectrum of animal models is available to develop and test such therapies and methods. However, since the pathomechanisms of MS are highly complex none of these models reflects the whole spectrum of MS and due to an artificial way of induction all these models have their limitation (Table 1). EAE reflects the autoimmune origin of MS and is probably the most frequently used model that already has been proven as extremely useful to develop/establish/test some of the currently available immunomodulating MS therapeutics (Gold et al., 2000, 2006; Constantinescu et al., 2011). Since multiple environmental factors such as Epstein-Barr Virus (EBV) infections were suggested to be strongly linked to the development of MS viral animal models, such as Semliki Forest virus (SFV), mouse hepatitis virus (MHV), or Theiler's murine encephalomyelitis virus (TMEV) demyelination models may be helpful to address this hypothesis (Suckling et al., 1978; Serafini et al., 2007; Drescher and Sosnowska, 2008; Lane and Hosking, 2010; Tsunoda and Fujinami, 2010). Genetic models are useful to study dysfunctions of myelination and axonal transport but they are not suitable to investigate remyelination. The assessment of remyelination is complicated in EAE and in virus mediated models due to concurrently occurring de- and remyelination. Thus, the evaluation of efficiency of remyelination-boosting factors might by limited. Another drawback of autoimmune-induced demyelination is the unpredictable localization of lesions that classically occur in the spinal cord. In the toxic animal models such as lysolecithin and ethidium bromide models, a focal myelin loss is induced by stereotactic injection of the toxic compound into the selected region of the CNS (Yajima and Suzuki, 1979; Woodruff and Franklin, 1999). Toxic models are commonly used to study remyelination since their kinetics are well predictable and the lesion site is known. Another plus of these models is a clear read-out of remyelination since significant improvements of remyelination can be gradually reflected by functional recovery of the animals. One of the major drawbacks of these models is the need of an exact stereotactic surgical procedure. Furthermore, the application of the toxin produces a stab wound that may complicate the molecular processes. In contrast, a systemic oral administration of the toxin cuprizone does not require complicated equipment/certain surgical skills and is represented by a relatively simple, but as well highly reproducible and reliable protocol. However, the question arises whether cuprizone treatment represents a model for MS. Since lesion induction is not immune-mediated it is quite different to MS. Thus, similar to other toxin-induced demyelination models, lesion induction is probably not relevant to MS although some authors suggest that demyelination primarily induced by oligodendroglial damage linked to mitochondrial dysfunctions, as seen in MS pattern III and IV lesions, is mimicked by the cuprizone model (Torkildsen et al., 2008; Kipp et al., 2009; Liu et al., 2010; Veto et al., 2010; Acs and Komoly, 2012; Kang et al., 2012). Moreover, since recent studies have shown that the de- and remyelination extend as well the cellular composition seems to differ between white and gray matter MS lesions (Bo et al., 2003; Albert et al., 2007; Van Horssen et al., 2007) the cuprizone model becomes increasingly attractive to study the underlying pathophysiological mechanisms of de- and remyelination to uncover regional differences (Gudi et al., 2009; Pott et al., 2009; Schmidt et al., 2013). Cuprizone treatment is a good model to study remyelination and to answer general neurobiological questions of glial reactions after a demyelinating insult (Skripuletz et al., 2011a, 2013; Zendedel et al., 2013). Furthermore, the cuprizone model allows to dissect the molecular mechanisms without the interference with the peripheral immune system, which is rather difficult in EAE. Frequently the model is used in parallel to EAE or other animal models to study treatment effects of substances that promote remyelination, such as benztropine, quentiapine fumarate, or antagonists of LINGO-1 (Mi et al., 2009; Deshmukh et al., 2013; Zhornitsky et al., 2013).

**Table 1:** **Advantages and disadvantages of different animal models available to study the broad spectrum of MS-relevant processes**.

**Induction**	**Animal models**	**Strength**	**Limitation**
Autoimmune	EAE [induced by CNS tissue; myelin peptides (MBP, PLP, MOG); adoptive transfer of myelin reactive T cells]	-is the most frequently used model to study autoimmune encephalitis in the CNS -mimics MS with respect to clinical symptoms and pathology -there is a broad spectrum of EAE induction protocols, allowing to study different aspects of MS -displays a relatively sensitive read-out system -is mainly useful to test putative immunosuppressive and neuroprotective drugs, to study the behavior of different T cells subtypes, mechanisms of neuronal damage and loss, reactions of resident glial cells and their interplay with peripheral immune cells, to investigate the role of different molecular factors on T cells activation and to study BBB dysfunctions	-an artificially induced sensitisation to myelin compounds -typically requires an application of adjuvant to activate the innate immune system -there is probably a different way of T cells priming as compared to MS -high complexity due to involvement of different cell types -high variety in susceptibility to EAE in dependence of strains, gender, species or even different animal colonies potentially leading to discrepancies in different EAE studies -does not reflect aspects of progressive MS -the assessment of remyelination is at least problematic since both de- and remyelination can proceed simultaneously -unpredictable localisation of lesions that classically occur in the spinal cord
Viral-autoimmune	Theiler's murine encephalomyelitis virus (TMEV); mouse hepatitis virus (MHV); Semliki Forest virus (SFV)	-supports the environmental compound (early infections) of MS aetiology -allows to study the phenomenon of epitope spreading -useful to study mechanisms of viral infection of neuronal and glial cells as well as a viral persistence -useful to study immune-mediated and virus-triggered demyelination but also to test different regenerative, neuroprotective, and immunosuppressive therapeutics	-the assessment of remyelination is at least problematic since both de-and remyelination can proceed simultaneously -some protocols require surgery -TMEV is time consuming and can be induced only in mice -C57BL/6 mice clear TMEV → difficulties in use genetically modified strains that are mostly development in the C57BL/6 background
Toxic	Cuprizone (systemic oral application)	-well reproducible, established model -predictable kinetics of de- and remyelination -clear detection/evaluation system -simple induction protocol -de- and remyelination take place in different regions and in both white and gray matter following different spatial and temporal pattern -can be induced in different rodents and strains -available as an acute and chronic demyelination model -useful to study remyelination and cellular behavior in the absence of peripheral immune cells	-an artificial way of demyelination induction due to an irreversible damage of mature oligodendrocytes via a toxin of unknown mode of action -T, B cells independent → only of limited relevance for MS and the development of immunomodulating drugs -BBB remains intact -clinical symptoms only purely reflect MS; no good clinical read-out -time consuming (acute model: 5–8 weeks; chronic: 12–16 weeks) -not useful to study spinal cord demyelination since demyelination occurs only in the brain
	Lysolecithin and Ethidium bromide (focal injection of a toxin)	-well reproducible, established model -predictable kinetics of de- and remyelination -well known lesion site can be induced in brain or spinal cord -useful to study remyelination and cellular behavior -useful to test putative immunosuppressive, neuroprotective, and especially regenerative agents	-an artificial way of demyelination induction due to an irreversible selective damage of myelin producing cells in the lysolecithin model. Ethidium bromide damages all nucleolus containing cells. -T, B cells independent → only of limited relevance for MS -tissue damage due to injection procedure -high complexity and difficulties in the assessment of remyelination in the spinal cord since different myelinating cells are involved in the remyelination
Genetic	Jimpy, Shiverer (shi), Rumpshaker mice	-consistency in myelination defects -useful to study dysfunctions of myelination, neuronal and glial behavior	-non inflammatory models -poor relevance for MS

## Lessons learned and practical suggestions

There are different protocols for the duration of cuprizone treatment, concentration of cuprizone used, and time points of investigations which leads to difficulties in the interpretation of the data. Many investigators feed C57BL/6 mice with 0.2% (w/w) cuprizone for 6 weeks to achieve complete demyelination. However, it was already described in the early work by the group of Matsushima and confirmed by others that complete demyelination of the CC occurs at week 5 of cuprizone feeding. Despite continued cuprizone exposure mature oligodendrocytes occur between week 5 and 6 and start subtle remyelination. When cuprizone is fed even longer these oligodendrocytes are damaged again and the myelin disappears after week 6. Thus, it should be kept in mind that remyelination does not strictly start with the end of cuprizone administration but all the necessary prerequisites are already made available before that. A recent study provided insights that even 2–3 weeks of cuprizone ingestion is sufficient to induce glial responses and de- and remyelination pattern similar to that described for 5–6 week treatment (Doan et al., 2013).

In summary, the following practical lessons can be learned from the studies published in the past 50 years:

1. Most studies in this model were performed in mice, but rats, guinea pigs, and hamsters are also susceptible to cuprizone.
2. Cuprizone is usually administrated mixed in the powdered rodent chow. Cuprizone containing pellets are also possible. However, since cuprizone is a heat sensitive compound, it is important to pay attention to temperature conditions when preparing the pellets.
3. The extent of cuprizone-induced demyelination is mouse strain specific. In the last two decades mice of the C57BL/6 background were mainly used for investigations and are thus the best-characterized strain. When using other strains the controls need to be characterized carefully.
4. Cuprizone-induced demyelination is age-, and in some strains also gender-dependent. Usually young (8–10 weeks) male C57BL/6 mice are used in the cuprizone model.
5. The dose of cuprizone needs to be adjusted in accordance to species, mouse strains, and age of animals. For young male C57BL/6 mice (8–10 weeks old) the cuprizone dose of 0.2% (w/w) is known to be well tolerated and reliable to induce a profound demyelination. When using other doses again the controls need to be well characterized since comparison to most published studies is not possible.
6. Demyelination in the cuprizone model is region-dependent. Most studies have investigated the medial CC where complete demyelination occurs after 5 weeks of 0.2% (w/w) cuprizone administration. We therefore, recommend to stop cuprizone after 5 weeks. However, complete demyelination in the cortex or the hippocampus is reached after 6 weeks of cuprizone feeding and thus the duration of treatment needs to be adjusted according to the region to be investigated. The spatiotemporal pattern of cuprizone-induced de- and remyelination is brain region specific and may even vary within a structure like the CC. Therefore, it is of great importance to always investigate a similar bregma level in all animals.
7. Remyelination is fast and robust after acute demyelination (cuprizone feeding for 5 weeks). Thus, in young mice differences may be picked up only early at 0.5 or 1 week after stopping cuprizone feeding since smaller effects may be compensated at later time points. After chronic demyelination (12 weeks) the remyelination capacity retains after withdrawal from the cuprizone diet but is strongly decreased.
8. Different myelin proteins are temporally individually degraded and re-expressed. Knowledge of this pattern is required to plan and interpret the experiments.
9. In order to generate sustainable conclusions it is important to investigate several time points and chose them carefully depending on the question to be answered.
10. Models using additional inhibitors of proliferation like rapamycin in order to better synchronize remyelination are not well characterized. Such substances also affect microglia and astrocytes the reaction of which has not been characterized so far. Thus, the system is even more artificial and the interpretation of results may be impossible because beside the oligodendrocyte damage there is interference with all other proliferating cells.

## Conclusion

**Figure 3:**
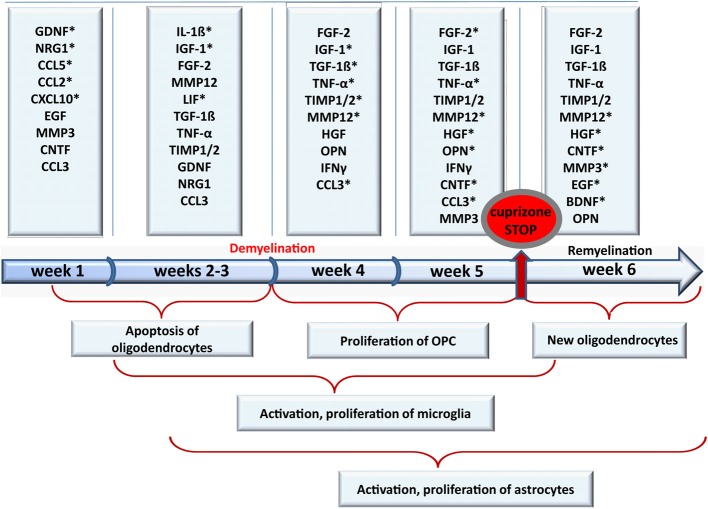
**Growth factors, cytokines, chemokines, and MMP expressed in the medial corpus callosum during cuprizone-induced de- and remyelination.** The expression (mostly mRNA expression) of different growth factors, cytokines, chemokines, and MMP were summarized from different studies where 0.2% (w/w) cuprizone was fed for 5 to 8–10-week old C57BL/6 mice (Mason et al., 2000a; McMahon et al., 2001; Selvaraju et al., 2004; Gudi et al., 2011; Skuljec et al., 2011; Buschmann et al., 2012; Voss et al., 2012; Skripuletz et al., 2013). The expression of various factors is linked to the cellular response during de- and remyelination. Asterisks marked the maximal expression of factors in certain weeks.

**Figure 4:**
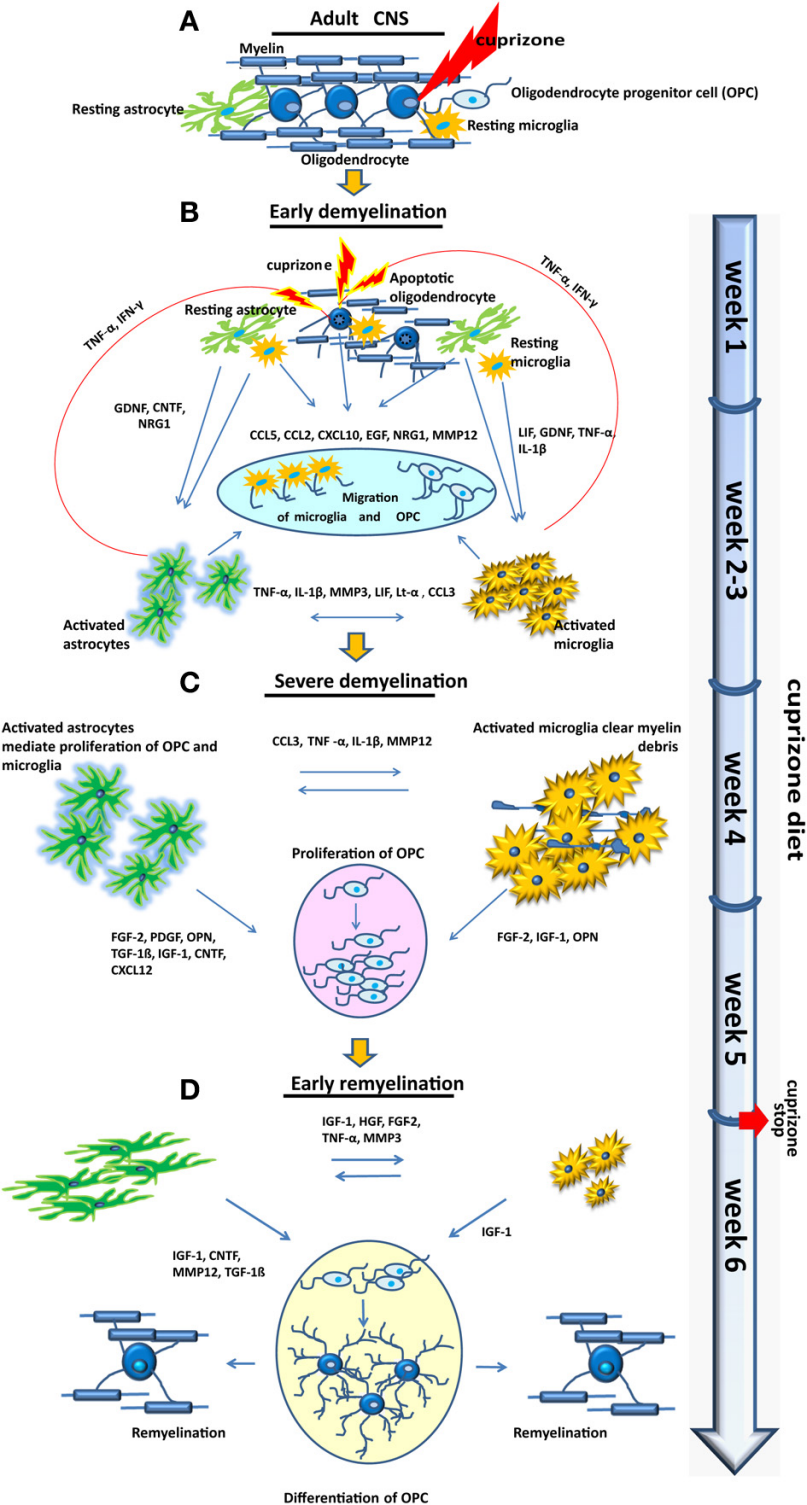
**Cellular and molecular response in the medial corpus callosum during cuprizone-induced de- and remyelination. (A)** Distribution of glial cells under normal conditions. **(B)** Weeks 1–3, “early demyelination.” Mature oligodendrocytes begin to undergo apoptosis already during the first week of cuprizone feeding. Microglia are highly motile cells. They may detect apoptotic oligodendrocytes and initiate together with astrocytes numerous inflammatory and probably also reparative processes. Various chemokines, cytokines, and growth factors, promoting attraction and activation of microglia and astrocytes but also migration of OPC are produced during the first week of cuprizone treatment. In the next 2 weeks of the “early demyelination” period inflammatory cytokines are already slightly up-regulated and maintain the inflammatory cycle by further promoting activation and proliferation of astrocytes and microglia but probably harming oligodendrocytes as well. Oligodendrocytes are almost completely depleted at week 3 of cuprizone treatment. **(C)** Weeks 3.5–5, “severe demyelination.” Activated microglia begin to clear myelin debris. Several MMP, chemokines, cytokines, and growth factors produced by activated astrocytes and microglia are supporting phagocytosis and promoting proliferation of OPC. At week 5 nearly all axons in the medial corpus callosum are demyelinated. **(D)** Weeks 5–6 “early remyelination.” In this week the amount of activated microglia begins to decline. Astrocytes are still activated but change their morphology. OPC differentiate and begin to remyelinate nude axons.

The cuprizone model is a well established and investigated paradigm to study de- and remyelination in rodents. It seems that the regeneration program initiated after the toxic insult via cuprizone proceeds in a highly controlled manner running sequences of tightly orchestrated events on molecular and cellular levels beginning with an activation of microglia and astrocytes as a response to the oligodendrocyte insult within the first 3 weeks of cuprizone feeding followed by a massive proliferation of all glial cells and myelin clearance between the third and fifth week, and onset of remyelination around week 5.5 (Figures 3, 4). There is growing evidence that in particular a limited inflammation may play a beneficial role in oligodendroglial regeneration and is even a preamble for successful remyelination. Activated microglia and astrocytes are sources of most factors. Microglia seem to be crucial for remyelination by promoting phagocytosis of myelin debris and production of factors regulating development of oligodendroglial cells. The role of astrocytes in the cuprizone model is still not completely elucidated. However, more and more studies emerge postulating astrocytes as a central cell type supporting a number of regenerative processes.

Although well characterized the cuprizone model lacks the autoimmune component of MS. Thus, it possesses only a limited spectrum of MS-relevant mechanisms in particular in lesion induction. However, it is useful to study T cells-independent de- and especially remyelination as well as basic myelin repair mechanisms, interaction of glial cells and their communications with axons. Since no animal model covers the complex pathomechanisms of human MS, the various animal models should be applied selectively or complementary to investigate MS-relevant processes and develop putative regenerative and protective treatments complementing immunomodulating therapies.

### Conflict of interest statement

The authors declare that the research was conducted in the absence of any commercial or financial relationships that could be construed as a potential conflict of interest.
